# Association of nonsteroidal anti-inflammatory drugs and aspirin use and the risk of head and neck cancers: a meta-analysis of observational studies

**DOI:** 10.18632/oncotarget.11239

**Published:** 2016-08-12

**Authors:** Lanhua Tang, Huabin Hu, Huai Liu, Chengzhu Jian, Hui Wang, Jin Huang

**Affiliations:** ^1^ Department of Oncology, Xiangya Hospital of Central South University, Changsha, China; ^2^ Department of Medical Oncology, The Sixth Affiliated Hospital of Sun Yat-Sen University, Guangzhou, China; ^3^ Department of Radiotherapy, Hunan Cancer Hospital and The Affiliated Cancer Hospital of Xiangya School of Medicine, Central South University, Changsha, China; ^4^ Key Laboratory of Translational Radiation Oncology, Changsha, China; ^5^ Department of Urology, The Second Xiangya Hospital of Central South University, Changsha, China

**Keywords:** head and neck cancer, nonsteroidal anti-inflammatory drugs, aspirin, chemoprevention, meta-analysis

## Abstract

**Purpose:**

Nonsteroidal anti-inflammatory drugs (NSAIDs), including aspirin, have emerged as the potential chemopreventive agents for a number of cancer types, however, previous studies of head and neck cancers (HNC) have yielded inconclusive results. We performed a meta-analysis of observational studies to quantitatively assess the association between NSAIDs use and the risk for HNC.

**Methods:**

We searched Pubmed, Embase, Google scholar, and Cochrane library for relevant studies that were published in any language, from January 1980 to April 2016. We pooled the odds ratio (OR) from individual studies and performed subgroup, heterogeneity, and publication bias analyses.

**Results:**

A total of eleven studies (eight case-control studies and three cohort studies), involving 370,000 participants and 10,673 HNC cases contributed to this meta-analysis. The results of these studies suggested that neither use of overall NSAIDs (OR=0.95; 95% CI, 0.81-1.11), aspirin (OR=0.93; 95% CI, 0.79-1.10), nor nonsteroidal NSAIDs (OR=0.92; 95% CI, 0.76-1.10) were associated with HNC risk. Similar nonsteroidal results were observed when stratified by HNC sites, study design, sample size, and varied adjustment factors. However, we found significant protective effect of ibuprofen (OR=0.85; 95% CI, 0.72-0.99) and long-term aspirin use (≧5years) (OR=0.75; 95% CI, 0.65-0.85) on HNC risk, with low heterogeneity and publication bias.

**Conclusions:**

Our meta-analysis results do not support the hypothesis that overall use of NSAIDs significant reduces the risk of HNC. Whereas, we cannot rule out a modest reduction in HNC risk associated with ibuprofen and long-term aspirin use.

## INTRODUCTION

Head and neck cancers (HNC) continues to be an important public health problem, with an estimated 700,000 new cases around the world in 2012 [1]. HNC includes a variety of cancers originating from different sites within the head and neck region, such as the oral cavity, oropharynx, hypopharynx and larynx. It is well documented that tobacco consumption and excessive alcohol drinking are independent major risk factors for the development of HNC [2–4]. Other possible risk factors include infection with human papilloma virus (HPV), poor oral hygiene, environmental carcinogens and genetic susceptibility [5–9]. Despite great advance in multidisciplinary treatment, approximately 30 - 50% patients with HNC survive over 5 years after initial diagnosis, and 15% - 20% patients will develop second primary malignancies after extensive therapy [10, 11]. Therefore, it is crucial that identifying potential chemopreventive measures other than tobacco and alcohol cessation should be further investigated.

Convincing laboratory evidence has emerged to demonstrate an association between chronic inflammation and cancer, which makes the anti-inflammatory drugs have emerged as the most potential chemopreventive agents [12–15]. Nonsteroidal anti-inflammatory drugs (NSAIDs), have received increasing attention because of their inhibitive effect on the cyclo-oxygenase (COX) enzymes, which may prevent synthesis of prostaglandins that stimulate growth and play a role in promoting carcinogenesis [16–19].

Several epidemiologic studies have observed use of aspirin and other NSAIDs have an association with reduced risk for cancers of the colon, stomach, prostate and breast [17, 20–22]. Even though some studies supported NSAIDs use significantly decrease the risk of HNC [11, 23, 24], while other studies did not show a consistent benefit [25, 26]. On the basis of the previous systematic review by Wilson et al. in 2011 [27], no definitive conclusion can be reached on NSAID/aspirin use and HNC risk. In addition, no meta-analysis and quantitative analysis was conducted due to the limited case number and heterogeneity of the studies identified. The purpose of the present study was to summarize all available evidence on this issue using a meta-analysis of observational studies.

## RESULTS

### Eligible studies

**Figure 1:**
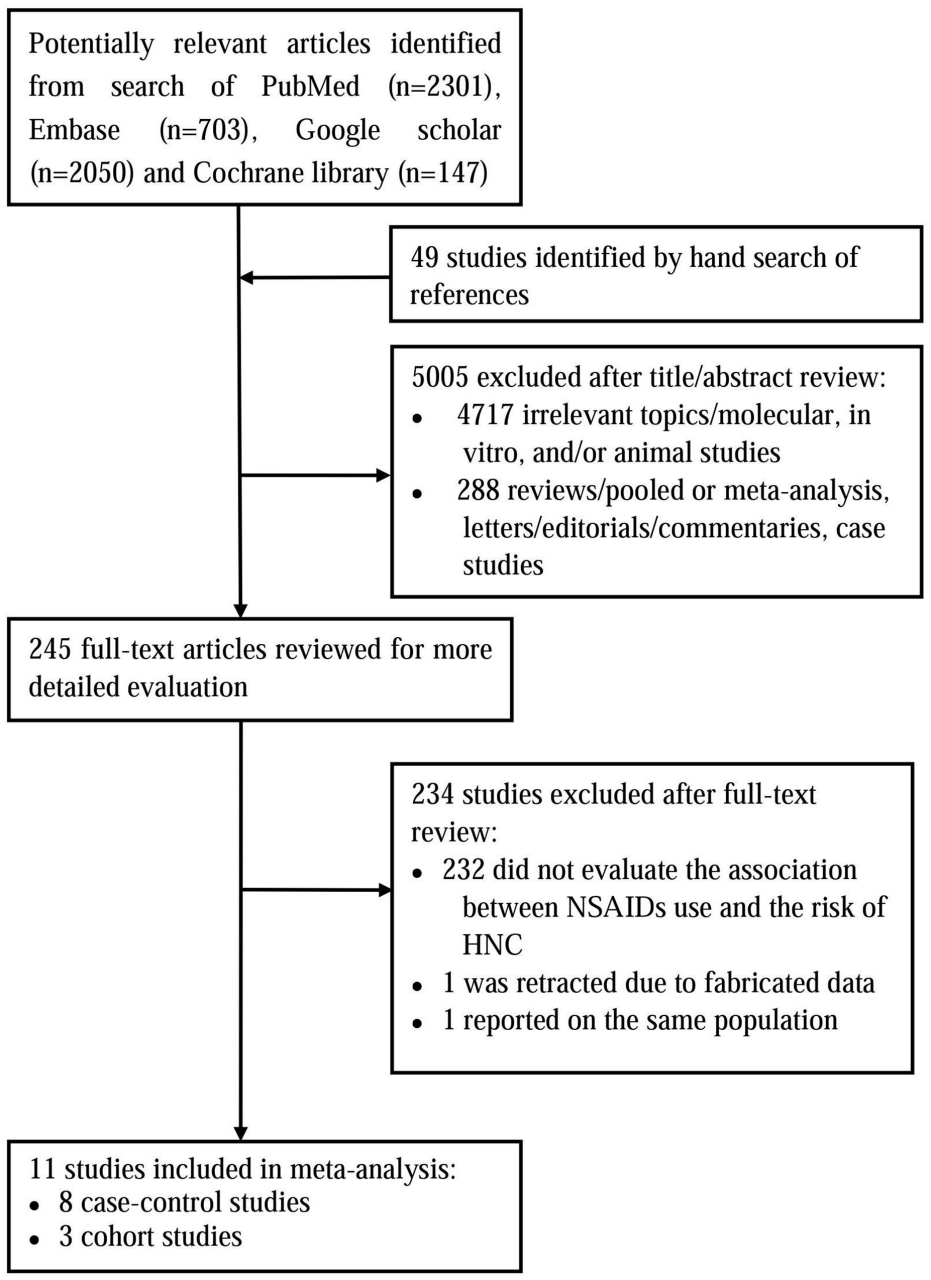
The flow diagram of search strategy

The overview of our search process was illustrated in Figure 1. Eleven studies met the predetermined criteria for inclusion, with three cohort studies [24, 28, 29], and eight case-control studies [11, 23, 25, 26, 30–33]. The number of HNC cases ranged from 71 to 2,745 in the case-control studies, and from 68 to 185 in the cohort studies. Three studies were conducted in the United States [11, 24, 31], two in Denmark [28, 29], two in United Kingdom [26, 32], two in Italy [23, 33], one in Sweden [30] and the other one was coordinated by the International Agency for Research on Cancer (IARC) in fourteen European centers (in ten countries) [25]. These eleven studies were published between 2003 and 2015. The range of enrollment periods for participants across studies was 1982-2013. Table 1 and Supplementary Table S1 listed the study characteristics and corresponding estimated OR with 95%CIs.

**Table 1:** Characteristics of studies included in the meta-analysis of NSAIDs use and the risk of HNC

Study	Study design	Study location	Period	HNC sites	Cases	Controls or cohort size	Type of drugs	OR (95%CI)	Adjustment for covariates
Bosetti et al, 2003	Case-control	Italy	1992 - 2000	Combined sites (oral, pharynx and larynx)	965	1779	Aspirin (at least once a week for more than 6 months)	0.86 (0.46 − 1.61)^a^	Age, gender, centre, educational, smoking, alcohol drinking
					935	1732	Long-term aspirin use (duration of use ≧ 5 years)	0.33 (0.13 − 0.82)	
Friis et al, 2003	Cohort	Denmark	1989 - 1995	Combined sites	68	29470	Aspirin (75, 100 or 150 mg)	1.36 (1.04 − 1.77)^b^	Age, gender
				Oral and oropharynx	48			1.30 (0.90 − 1.70)	
				Larynx	20			1.50 (0.90 − 2.40)	
Rosenquist et al, 2005	Case-control	Sweden	2000 - 2004	Oral and oropharynx	132	320	Aspirin (75-150 mg/day)	1.00 (0.60 − 1.70)^a^	Smoking, alcohol drinking
Friis et al, 2006	Cohort	Denmark	1991 - 2002	Oral (month and tongue)	185	169589	NA-NSAIDs (≧ 2 prescriptions)	1.20 (1.00 − 1.60)	Age, gender
Jayaprakash et al, 2006	Case-control	United States	1982 - 1998	Combined sites	529	529	Aspirin (ever used)	0.75 (0.58 − 0.96)	Age, gender, smoking, alcohol drinking
				Oral cavity	169	169		0.73 (0.51 − 1.05)	
				Nasopharynx	22	22		0.88 (0.36 − 2.15)	
				Oropharynx	102	102		0.68 (0.44 − 1.05)	
				Larynx	141	141		0.89 (0.58 − 1.35)	
				Hypopharynx	31	31		0.77 (0.35 − 1.66)	
				Combined sites	313	289	Long-term aspirin use (duration of use ≧ 10 years)	0.65 (0.52 − 0.82)^c^	
Ahmadi et al, 2010	Case-control	United States	2003 - 2007	Combined sites	71	71	Any NSAIDs (ever used)	0.31 (0.11 − 0.88)	Educational, marital status
					44	47	Any NSAIDs (daily)	0.14 (0.04 −0.54)	
					25	25	Aspirin (daily)	0.15 (0.02 − 1.30)	
Macfarlane et al, 2012	Case-control	European	NR	Combined sites	1779	1993	Aspirin (at least once a week for one year)	0.92 (0.78 − 1.09) ^b^	Age, gender, BMI, educational, smoking, alcohol drinking, fruit consumption
				Oral cavity	510	1993		1.04 (0.76 − 1.41)	
				Oropharynx	474	1993		1.05 (0.76 − 1.46)	
				OP NOS	112	1993		1.29 (0.70 − 2.35)	
				Larynx	670	1993		0.74 (0.54 − 1.01)	
				Hypopharynx	183	1993		0.53 (0.28 − 1.02)	
				Combined sites	1588	1736	Long-term aspirin use (duration of use ≧ 5 years)	0.78 (0.58 − 1.05) ^d^	
Wilson et al, 2013	Cohort	United States	1993 - 2001	Combined sites	316	141718	Aspirin (regular use)	0.78 (0.62 − 0.98)	Age, gender, BMI, smoking
					315	141550	Aspirin (daily)	0.85 (0.65 − 1.11)	
					316	141718	Ibuprofen (regular use)	0.97 (0.75 − 1.27)	Age, gender, BMI, smoking, aspirin use
Macfarlane et al, 2014	Case-control	United Kingdom	1996 - 2010	Combined sites	2392	7165	Aspirin (≧ 1 prescriptions)	0.93 (0.76 − 1.15)	Age, gender, carstairs deprivation category, coronary heart disease, stroke
					2034	6094	Long-term aspirin use (time between last and first prescription ≧ 5.4 years)	0.85 (0.70 − 1.04)	
					2392	7165	NA-NSAIDs (≧ 1 prescriptions)	0.82 (0.70 − 0.96)	
Becker et al, 2015	Case-control	United Kingdom	1995 - 2013	Combined sites	2238	13488	Aspirin (≧ 50 prescriptions)	1.21 (0.97 − 1.51)	BMI, smoking, alcohol drinking
					1932	11657	Ibuprofen (≧ 6 prescriptions)	0.78 (0.64 − 0.96)	
Di Maso et al, 2015	Case-control	Italy	1992 -2008	Nasopharynx	198	592	Aspirin (at least one aspirin a week for more than 6 months)	0.24 (0.07 −0.87)	Age, gender, area of residence, smoking, period of interview, years of education, occupation

Abbreviations: BMI, body mass index; CI, confidence interval; F, female; HNC, Head and Neck Cancers; M, male; NR, not reported; NA-NSAIDs, non-aspirin nonsteroidal anti-inflammatory drugs; NSAIDs, nonsteroidal anti-inflammatory drugs; OP NOS, Oral, pharynx not otherwise specified; OR, odds ratio.

a Cited from a systematic review by Wilson et al.;

b Pooled from all cancer sites except for esophagus;

c Pooled from the ORs of 10-20 years, 21-40 years and ≧40 years;

d Pooled from the ORs of 5-9 years and ≧10years.

### Quality of study methodologies

Table 2 showed the quality of study methodology included in the meta-analysis. The range of quality scores was 5 - 8; the average score was 6.7. The average scores of cohort studies and case–control studies were 7.7 and 6.4, respectively, which suggests a reasonable good quality of the cohort and case–control studies.

**Table 2:** Methodological quality of included studies based on the Newcastle–Ottawa Scale

Cohort studies (n=3)	Representa-tiveness of the exposed cohort	Selection of the unexposed cohort	Ascertainment of exposure	Outcome of interest not present at start of study	Control for Important factor or additional factor	Assessment of outcome	Follow-up long enough for outcomes to occur ^a^	Adequacy of follow-up of cohorts	Total (0-9)
Friis et al, 2003	★	★	★	★	★	★	★	★	8
Friis et al, 2006	★	★	★	★	★	★	★	★	8
Wilson et al, 2013	★	★	-	★	★	★	★	★	7

Case-control studies (n=7)	Adequate definition of cases	Representa-tiveness of cases	Selection of contros	Definition of controls	Control for important factor or additional factor	Ascertainment of exposure	Same method of ascertainment for subjects	Nonresponse Rate ^b^	Total (0-9)
Bosetti et al, 2003	-	★	★	★	★★	-	★	-	6
Rosenquist et al, 2005	★	★	★	★	★	-	★	-	6
Jayaprakash et al, 2006	★	★	★	★	★★	-	★	-	7
Ahmadi et al, 2010	★	★	★	★	-	-	★	-	5
Macfarlane et al, 2012	★	★	★	★	★★	-	★	-	7
Macfarlane et al, 2014	★	★	★	★	★	★	★	-	7
Becker et al, 2015	★	★	★	★	★	★	★	-	7
Di Maso et al, 2015	★	★	-	★	★	-	★	★	6

a A cohort study with a follow-up time > 5 years was assigned one star;

b Same rate for both groups was assigned one star.

### Overall use of NSAIDs and the risk for HNC

**Figure 2:**
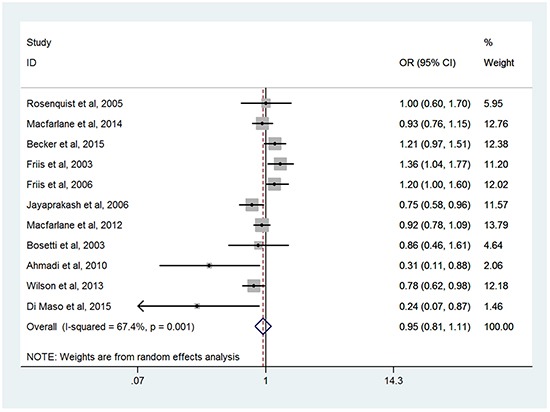
Forest plots of meta-analysis of overall NSAIDs use and the risk of HNC

Figure 2 illustrated the forest plot of ORs estimates with 95%CIs from individual studies and overall meta-analysis of all eleven studies [11, 23-26, 28-33]. The overall summary ORs demonstrated no significant association between overall NSAIDs use and the risk of HNC (OR=0.95; 95%CI, 0.81-1.11), with statistical heterogeneity among studies (*P*=0.001; *I^2^*=67.4%). The tests for funnel plot asymmetry by Begg's test and Egger's test identified no publication bias (Begg's test, *P* = 0.276; Egger's test, *P* = 0.229).

### Subgroup analyses

**Figure 3:**
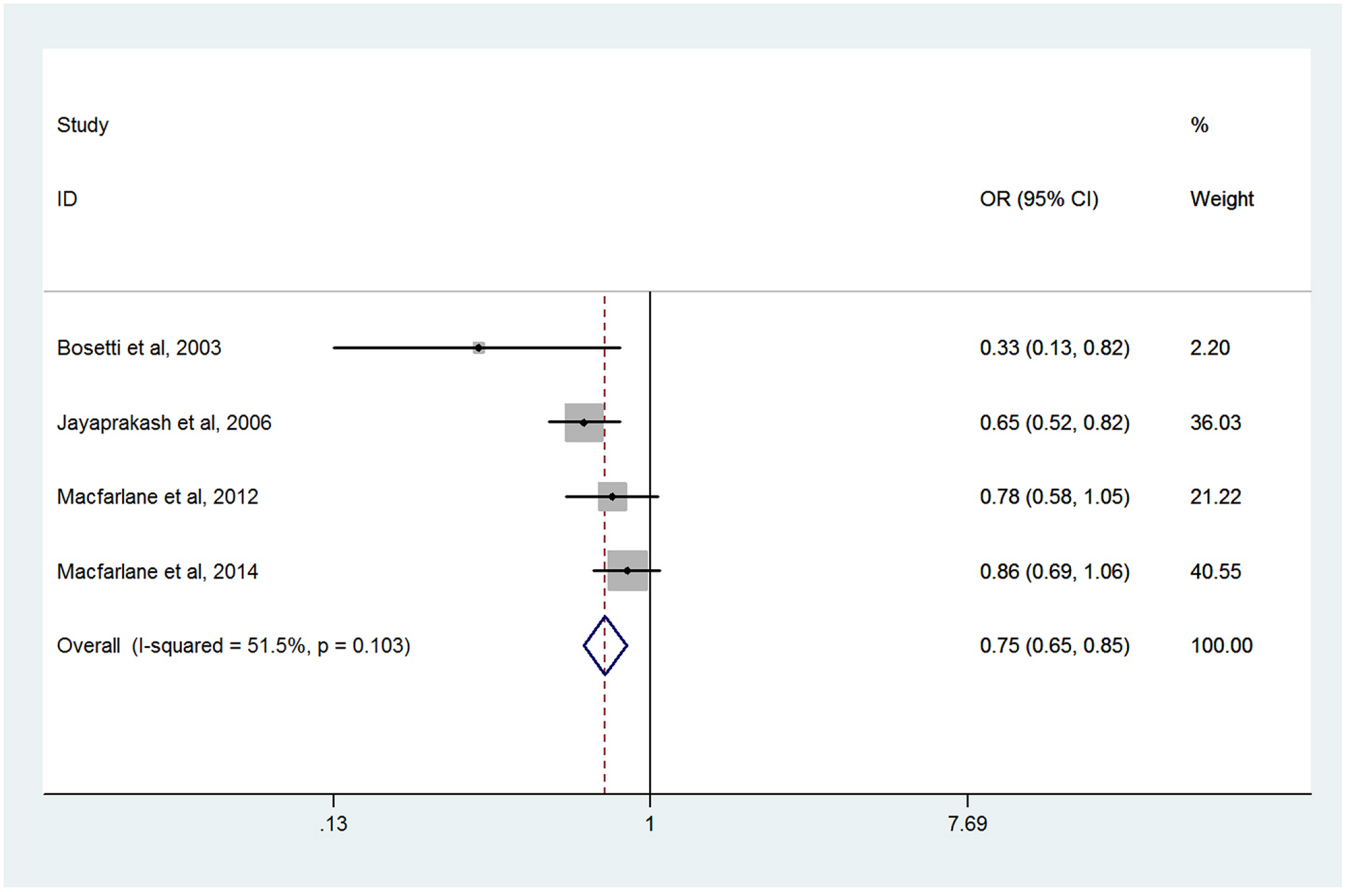
Forest plots of meta-analysis of long-term usage of aspirin and the risk of HNC

Table 3 showed the associations between NSAIDs use and the risk of HNC in subgroup meta-analyses by various factors. The subgroup analyses were conducted on the basis of different types of NSAIDs use. There was no significant association between use of aspirin [11, 23–26, 28, 30–33] or non-aspirin nonsteroidal anti-inflammatory drugs (NA-NSAIDs) [24, 26, 29, 32] and the risk of HNC, with OR of 0.93 (95%CI, 0.79-1.10) and OR of 0.92 (95%CI, 0.76-1.10), respectively. For the two studies evaluated exposure to ibuprofen and the risk of HNC [24, 32], a significant protective effect was observed (OR = 0.85; 95%CI, 0.72-0.99). We also found long- term usage of aspirin (≧5 years) has been associated with a significant 25% reduction in HNC risk from four studies [11, 23, 25, 26] (OR=0.75; 95%CI, 0.65-0.85) (Figure 3), and statistical heterogeneity was not detected.

**Table 3:** Associations between NSAIDs use and the risk of HNC in subgroup meta-analyses

Studies groups	No. of Studies	Summary OR (95% CI)	Homogeneity		Model used	Publication Bias	
			*P*-value	*I*^2^ (%)		Begg's*P-*value	Egger's *P-*value
**Type of drugs use**							
Aspirin	10	0.93 (0.79 − 1.10)	0.002	65.0%	Random-effects	0.474	0.255
NA-NSAIDs	4	0.92 (0.76 − 1.10)	0.026	67.7%	Random-effects	0.734	0.342
Ibuprofen	2	0.85 (0.72 − 0.99)	0.199	39.5%	Fixed-effects	1.000	NA
**Long-term aspirin use**	4	0.75 (0.65 − 0.85)	0.103	51.5%	Fixed-effects	0.308	0.313
**HNC sites**							
Oral and oropharynx	5	1.01 (0.85 − 1.20)	0.098	43.9%	Random-effects	0.230	0.139
Larynx	3	0.96 (0.65 − 1.42)	0.059	64.7%	Random-effects	0.296	0.253
Hypopharynx	2	0.62 (0.38 − 1.01)	0.469	0.0%	Fixed-effects	1.000	NA
Nasopharynx	2	0.50 (0.14 − 1.76)	0.099	63.2%	Random-effects	1.000	NA
**Study design**							
Case-control	8	0.89 (0.74 − 1.07)	0.017	59.1%	Random-effects	0.108	0.130
Cohort	3	1.08 (0.77 − 1.51)	0.003	82.4%	Random-effects	0.296	0.446
**Sample sizes**							
≧ 1000	8	0.99 (0.85 − 1.15)	0.003	67.1%	Random-effects	0.711	0.897
< 1000	3	0.47 (0.18 − 1.27)	0.031	71.1%	Random-effects	0.296	0.059
**Exposure source**							
Questionnaire	6	0.82 (0.73 − 0.92)	0.090	47.5%	Random-effects	0.060	0.040
Prescription database	4	1.14(1.02 – 1.28)	0.118	48.9%	Fixed-effects	0.308	0.134
**Adjustments for age**							
Yes	8	0.93 (0.79 − 1.11)	0.003	68.0%	Random-effects	0.711	0.478
No	3	0.89 (0.51 −1.53)	0.039	69.3%	Random-effects	0.296	0.233
**Adjustments for gender**							
Yes	8	0.93 (0.79 − 1.11)	0.003	68.0%	Random-effects	0.711	0.478
No	3	0.89 (0.51 −1.53)	0.039	69.3%	Random-effects	0.296	0.233
**Adjustments for smoking**							
Yes	7	0.88 (0.73 − 1.06)	0.022	59.6%	Random-effects	0.230	0.382
No	4	1.05 (0.78 − 1.40)	0.011	73.2%	Random-effects	1.000	0.454
**Adjustments for alcohol**							
Yes	5	0.95 (0.79 −1.14)	0.083	51.5%	Random-effects	1.000	0.908
No	6	0.91 (0.69 −1.21)	<0.001	77.5%	Random-effects	1.000	0.229
**Adjustments for BMI**							
Yes	3	0.95 (0.76 − 1.20)	0.023	73.6%	Random-effects	1.000	0.939
No	8	0.92 (0.72 − 1.17)	0.002	69.4%	Random-effects	0.386	0.164

Abbreviations: BMI, body mass index; CI, confidence interval; HNC, Head and Neck Cancers; NA, not available; NA-NSAIDs, non-aspirin nonsteroidal anti-inflammatory drugs; NSAIDs, nonsteroidal anti-inflammatory drugs; OR, odds ratio.

There are six studies provided results on the effect of NSAIDs for specific HNC sites. However, NSAIDs use was not associated with a reduced risk for cancer of oral and oropharynx [11, 25, 28–30] (OR=1.01; 95%CI, 0.85 − 1.20), larynx [11, 25, 28] (OR=0.96; 95%CI, 0.65- 1.42), hypopharynx [11, 25] (OR=0.62; 95%CI, 0.38 − 1.01) and nasopharynx [11, 33] (OR=0.50; 95%CI, 0.14 − 1.76).

To examine consistency across varying study designs with different potential biases, we stratified data into subgroups on the basis of study design. The summary ORs were 0.89 (95%CI: 0.74 − 1.07) pooled from eight case-control studies [11, 23, 25, 26, 30–33] and 1.08 (95%CI: 0.77 − 1.51) across three cohort studies [24, 28, 29].

The impact of sample size on risk estimates was assessed. The summary ORs were 0.99 (95%CI: 0.85 − 1.15) in eight studies [11, 23–26, 28–32] with relatively large sample size (≧1,000) and 0.47 (95% CI: 0.18 − 1.27) from the other three studies with the sample size less than 1,000 [30, 31, 33].

Recorded prescription database, self-administered questionnaires and standardized interviews were used to obtain information on NSAIDs exposure. The summary ORs were 0.82 (95%CI: 0.73 − 0.92) pooled from six questionnaire-based studies [11, 23–25, 31, 33] and 1.14 (95%CI: 1.02 − 1.28) from four studies based on prescription [26, 28, 29, 32]. These results suggested a significant protective effect was observed only in studies based on questionnaire.

In subgroup analyses by varied adjustment factors including age, gender, smoking, alcohol drink and body mass index (BMI), the association between NSAIDs use and the risk of HNC were non-significant in all strata (Table 2).

## DISCUSSION

The findings from this meta-analysis of eleven observational studies, including 370,000 participants and 10,673 HNC cases, did not indicate overall NSAIDs use was significantly associated with a decreased risk for HNC.

When stratified by type of drugs, there was no association between use of aspirin or NA-NSAIDs and the risk of HNC. However, we observed a significant risk reduction of 15% in HNC risk for users of ibuprofen alone (OR = 0.85; 95%CI, 0.72-0.99). It was suggested that different types of NSAIDs might have different effects due to biological mechanisms [34, 35]. Andrews et al. had demonstrated that ibuprofen was more effective at reducing cancer cell growth and survival across a variety of cancer cell lines compared to other NSAIDs [36, 37], which may explain our findings.

It is widely accepted that any potential protective effects of NSAIDs use against cancers are likely to involve a considerable duration [38]. Previous large randomized trials and cohort studies showed long-term use of aspirin and other NSAIDs have almost consistently been associated with a stronger reduced risk of colorectal cancer [39–41]. The time-risk relations are similar to those described for colorectal cancer, our results also observed a significant association between long-term aspirin use and the risk of HNC (OR=0.78; 95%CI, 0.67-0.92).

A significant preventive effect of NSAIDs use on HNC risk was observed in questionnaire-based studies but not in prescription-based studies. Studies that use prescription databases have their own shortcomings. Generally, no data were available regarding the use of over-the-counter medications including aspirin and NSAIDs, which will have underestimated exposure to these drugs. However, results from questionnaire-based studies should be interpreted cautiously for recall bias or selection bias. If the relatively healthy aspirin users were more likely to participate in the study than non-users, and an overestimate inverse association could be observed.

The previous systematic review including 2 cohort studies [28, 29] and 3 case-control studies [11, 23, 30], conducted by Wilson et al. also suggested no definitive conclusion can be reached on the preventive effect of NSAIDs on HNC risk [27]. The strengths of our meta-analysis were as follows: First, our present study included sufficient cases and quantitatively analyzed the effect of NSAIDs/aspirin using a detailed meta-analysis of eleven observational studies; Second, we were more capable to investigate potentially different effects on risk by the type and duration of NSAIDs use. As mentioned in the conclusion of Wilson's systematic review, aspirin may protect against HNC. We not only found a significant risk reduction in HNC risk for long-term aspirin but also ibuprofen user; Third, we performed the publication bias and more subgroup analyses, which reinforce our confidence in the validity of the conclusion.

There were some potential limitations that have to be considered when interpreting these results. First, this meta-analysis is based on observational studies, which are more susceptible to recall and selection biases, and could result in an underestimation or an overestimation of the true effect. However, It may be unfeasible to evaluate the long-term protective effects of NSAID from randomized clinical trials due to the large sample size required; Second, we did not take into account possible interactions with other drugs due to absence of data. Use of NSAIDs are often associated with other drugs use, which could have concealed a possible association with NSAIDs [42]. Third, none of the studies included adjusted the analyses for HPV infection, which has been shown to have an etiological role in HNC as well as smoking and heavy alcohol drink, and there was some evidence to suggest an up-regulation of COX-2 in HPV-infected tissues [43, 44]. Fourth, HNC are a heterogeneous group of neoplasms, and we conducted subgroup analyses separately for specific HNC sites, however no significant risk reduction was found; Fifth, the possibility of publication bias is always a concern in meta-analyses of published studies. This could bias the results of this review if negative studies were less likely to be published. In our meta-analysis, a significant publication bias in the subgroup of long-term aspirin use was observed from the Egger's test (P=0.055) but not the Begg's test (P=0.308). We consider the discrepancy was due to the small number of studies included.

In conclusion, our meta-analysis does not support the hypothesis that overall use of NSAIDs significant reduces the risk of HNC. Whereas, we cannot rule out a modest reduction in HNC risk associated with ibuprofen and long-term aspirin use. Further large-scale robust studies are required, in particular, those evaluating the duration of aspirin use that may be take a protective effect.

## MATERIALS AND METHODS

### Publication search

This meta-analysis was conducted according to the meta-analysis of Observational Studies in Epidemiology (MOOSE) Guidelines [45]. We systematically searched Pubmed, Embase, Google scholar, and Cochrane library for manuscripts that mentioned the relationship between the use of aspirin and NSAIDs and the risk of HNC without language restriction, from January 1980 to April 2016. Our search terms consisted of three main components, head and neck (head and neck OR oral OR oropharynx OR hypopharynx OR larynx OR upper aerodigestive tract) AND disease (cancer OR neoplasms OR carcinoma) AND the exposure factor (aspirin OR NSAIDs OR ibuprofen OR naproxen OR indomethacin OR meloxicam OR valdecoxib OR celecoxib OR rofecoxib). We also reviewed the reference lists of articles with information on the topic to retrieve additional pertinent studies. If necessary, we attempted to contact the authors if we required additional information.

### Study selection

Studies that met the following criteria were eligible for inclusion: (1) use a case–control or cohort study design; (2) evaluate the association between NSAIDs use and the risk of HNC; (3) provided the odds ratio (OR) or relative risk (RR) with corresponding confidence interval (CI) or sufficient data to calculate them. When the same author reported results obtained from the same population in more than one publication, only the most recent report, or the most complete one, was included in the analysis. Data from review articles, case reports, abstracts, and letters were not included.

### Data extraction and quality assessment

Two investigators (Lanhua Tang and Huabin Hu) extracted the following information from each eligible studies independently: the last name of the first author, year of publication, study design, country where the study was performed, enrollment periods, HNC sites, sample size (numbers of case patients and control subjects), types of NSAIDs use, the source of NSAIDs exposure information, the source of HNC diagnosis, the ORs or RRs with corresponding 95%CI and adjustment for covariates. Differences in data extraction were resolved by consensus, referring back to the original article.

The methodological quality of the included studies was assessed using the 9-star Newcastle–Ottawa scale for quality of nonrandomized studies in meta-analyses [46]. The Newcastle-Ottawa Scale consists of eight items, which are categorized three categories: selection (one star each), comparability (up to two stars), and exposure/outcome (one star each). A “star” presents a “high-quality” choice of individual study.

### Statistical analysis

Odds ratio (OR) was used as a measure of the association between use of NSAIDs and risk of HNC. Because the absolute risk of HNC is low, the OR in case–control studies was considered reasonable approximations of the corresponding rate ratios in cohort studies [47]. The heterogeneity of the estimators of OR was tested by Cochran's Q test at the *P* <0.10 level of significance [48]. We also calculated the quantity *I*^2^ that describes the percentage variation across studies that is attributed to heterogeneity [49, 50]. When significant heterogeneity was found, the random-effects model with the DerSimonian-Laird method was used for meta-analysis [51]. Otherwise, the fixed-effects model with Mantel–Haenszel method was adopted [52]. Publication bias was evaluated using the Begg's adjusted rank correlation test [53], and the Egger's regression asymmetry test [54]. *P* <0.10 was considered to represent statistically significant publication bias.

When study reported results separately for aspirin and non-aspirin NSAIDs (NA-NSAIDs), to avoid double counting of the cases, we included only the results for aspirin in the overall summary estimate. Use of NSAIDs has the definition as follows:“overall use” was all the reported intake levels of NSAIDs use,“long-term use” was defined the duration of NSAIDs use or the time between last and first prescription more than 5 years. We only calculated the results for combined sites of HNC in the overall estimate, if studies had results for specific site and combined sites of HNC.

Analysis was performed using the STATA version 11.0 (Stata Corporation, College Station, Texas).

## SUPPLEMENTARY TABLE


