# Supplementary material for: Association of nonsteroidal anti-inflammatory drugs and aspirin use and the risk of head and neck cancers: a meta-analysis of observational studies
Source: Oncotarget. 2016 Aug 12;7(40):65196–207. doi: 10.18632/oncotarget.11239 (PMC5323148; doi:10.18632/oncotarget.11239)
Supplement: Supplementary file 1 [file oncotarget-07-65196-s001.pdf]

Association of nonsteroidal anti-inflammatory drugs and aspirin use and the risk of head and neck cancers: a meta-analysis of observational studies

SUPPLEMENTARY TABLE

Supplementary Table S1: Characteristics of studies included in the meta-analysis of NSAIDs use and the risk of HNC

Study	Study design	Exposure source	Source of HNC diagnosis	Mean or range of age (years)
Bosetti et al, 2003	Case-control	Questionnaire	NR	Range: 22-79 (case), 20-79 (control)
Friis et al, 2003	Cohort	Prescription database	Danish Cancer Registry	Mean: 70
Rosenquist et al, 2005	Case-control	Standardized interview	Hospital diagnosis	Range: 53-87
Friis et al, 2006	Cohort	Prescription database	Danish Cancer Registry	Range: ≧ 16
Jayaprakash et al, 2006	Case-control	Questionnaire	RPCI tumor registry and diagnostic index	Mean: 61 (case), 61 (control)
Ahmadi et al, 2010	Case-control	Questionnaire	Hospital diagnosis	Mean: 56 (case), 57 (control)
Macfarlane et al, 2012	Case-control	Questionnaire	Hospital diagnosis	NR
Wilson et al, 2013	Cohort	Questionnaire	Hospital diagnosis	Range: 55-74
Macfarlane et al, 2014	Case-control	Prescription database	Primary Care Clinical Informatics Unit Database	Mean: 66 (case)
Becker et al, 2015	Case-control	Prescription database	UK-based Clinical Practice Research Datalink	Mean: 62 (case), 62 (control)
Di Maso et al, 2015	Case-control	Questionnaire	Hospital diagnosis	Range: 18-76 (case), 19-76 (control)

Abbreviations: HNC, Head and Neck Cancers; NR, not reported.
